# Protein complex detection with semi-supervised learning in protein interaction networks

**DOI:** 10.1186/1477-5956-9-S1-S5

**Published:** 2011-10-14

**Authors:** Lei Shi, Xiujuan Lei, Aidong Zhang

**Affiliations:** 1 Computer Science & Engineering Department, State University of New York at Buffalo, Buffalo, NY, USA; 2 Computer Science Department, Shanxi Normal University, Xi’an, Shanxi Province, 710062, China

## Abstract

**Background:**

Protein-protein interactions (PPIs) play fundamental roles in nearly all biological processes. The systematic analysis of PPI networks can enable a great understanding of cellular organization, processes and function. In this paper, we investigate the problem of protein complex detection from noisy protein interaction data, i.e., finding the subsets of proteins that are closely coupled via protein interactions. However, protein complexes are likely to overlap and the interaction data are very noisy. It is a great challenge to effectively analyze the massive data for biologically meaningful protein complex detection.

**Results:**

Many people try to solve the problem by using the traditional unsupervised graph clustering methods. Here, we stand from a different point of view, redefining the properties and features for protein complexes and designing a “semi-supervised” method to analyze the problem. In this paper, we utilize the neural network with the “semi-supervised” mechanism to detect the protein complexes. By retraining the neural network model recursively, we could find the optimized parameters for the model, in such a way we can successfully detect the protein complexes. The comparison results show that our algorithm could identify protein complexes that are missed by other methods. We also have shown that our method achieve better precision and recall rates for the identified protein complexes than other existing methods. In addition, the framework we proposed is easy to be extended in the future.

**Conclusions:**

Using a weighted network to represent the protein interaction network is more appropriate than using a traditional unweighted network. In addition, integrating biological features and topological features to represent protein complexes is more meaningful than using dense subgraphs. Last, the “semi-supervised” learning model is a promising model to detect protein complexes with more biological and topological features available.

## Background

High-throughput assay methodologies, such as microarrays and mass spectrometry, have resulted in the rapid growth of protein data sets, the analysis of which can potentially yield insights into the mechanisms of human diseases and the discovery of new therapeutic interventions [1][2]. Systematic analysis of the underlying relationships in these protein data sets can potentially provide useful insights into roles of proteins in biological processes [3][4][5][6].

PPI data sets provide us the good opportunity to systematically analyze the structure of a large living system and also allow us to use it to understand essential principles like essentiality, genetic interactions, functions, functional modules, protein complexes and cellular pathways [7]. Cellular functions and biochemical events are coordinately carried out by groups of proteins interacting with each other in functional modules, and the modular structure of complex networks is critical to functions [8]. Identifying such protein complexes in PPI networks is very important for understanding the structure and function of these fundamental cellular networks. Therefore, developing an effective computational approach to identify those protein complexes should be highly challenging but indispensable.

However, protein complexes are likely to overlap and the interaction data are very noisy. It is a great challenge to effectively analyze the massive data for biologically meaningful protein complex detection. Since most proteins form macromolecular complexes involving two or more proteins to perform biological functions, many people assume protein complexes should be dense subgraphs. Thus some graph clustering based algorithms could be applied to it. Molecular Complex Detection (MCODE) [9] is the first computational method to detect protein complexes from PPI networks. MCODE first identifies densely connected subgraphs and then uses another post-processing to filter non-dense subgraphs and generate overlapping clusters. Later, Spirin and Mirny [10] proposed a clique based algorithm, which exhaustively searches all the full cliques as protein complexes in the network. Since using clique is too constrained, they modified it by applying the Super-Paramagnetic Clustering (SPC) and a Monte Carlo (MC) simulation for the same purpose. Instead of adopting the over-constraining full cliques as the basis for protein complexes, Li *et al.*[*11*] devised an LCMA algorithm (Local Clique Merge Algorithm) that adopts a local clique merging method as an attempt to address the current incompleteness limitation of protein interaction data. Amin *et al.*[*12*] proposed a cluster periphery-tacking algorithm (DPCLus) to detect protein complexes by keeping track of the periphery of a detected cluster. Chua *et al.*[*13*] proposed an algorithm called PCP (ProteinComplexPrediction) for complex prediction, which utilized the filtered PPI network by FS-weight [14], clique finding and merging techniques. Ucar *et al.*[*15*] developed a refinement method, which uses hub protein duplication strategy to detect dense subgraphs in scale-free PPI networks with multi-functional hub proteins assigned to multiple clusters. Adamcsek *et al.*[*16*] proposed a CFinder algorithm to find complexes in the PPI networks. CFinder detects k-cliques as modules and then merges modules by calculating their similarities. Mete [17] extended the density-based clustering method DBSCAN [18] and used it in the PPI networks. SCAN first forms a cluster by a core node then iteratively merges the neighboring nodes one by one. Finally, the detected clusters are formed to become the predicted protein complexes.

The previous methods are suffering from a serious problem, that is, they all assume protein complexes as dense subgraphs. As Qi *et al.*[*19*] pointed out, not all protein complexes are clique-oriented and there are quite a large amount of protein complexes with shapes like star-shape or other forms. In this paper, we will solve the problem from another perspective, redefining the properties and features for protein complexes and using a semi-supervised learning method to build a model to detect those hidden protein complexes in the scale-free PPI networks. First, we choose several biological and topological features to represent the protein complexes. Then, we use the “semi-supervised” mechanism to recursively train the neural network and obtain the optimized parameters for the model. Last, we use the neural network to detect the protein complexes in the protein interaction network.

The paper is organized as follows. First, we identify the difficulties of the problem. Second, we propose some favorable properties for protein complexes. Third, we propose the multi-layer neural network. Fourth, we conduct extensive experiments to verify the effectiveness of the proposed method. Finally, we conclude the paper and propose the future work.

## Challenges in protein complex detection

Through extensive observations, we found the following problems are the keys to detect protein complexes in the PPI networks.

• Protein interaction data are very noisy. Since a clustering method is based on the protein protein interactions in the graph, more reliable those interactions are, more accurate the clustering result will be. From the previous works [14][20][21], using a weighted and filtered graph instead of traditional unweighted graph to represent a PPI network is proven to be an effective way. Then the problem becomes how to obtain the reliable protein protein interactions in PPI data. Here we are using GO (Gene Ontology) to obtain the similarity between different proteins in the network and build a weighted graph with a setup threshold.

• Proteins may participate in multiple protein complexes. Therefore, protein complexes may overlap with each other. These overlaps correspond to proteins’ participation in multiple pathways and the crosstalk between different biological modules. Thus, the traditional paradigm for clustering and putting each protein into one single cluster doesn’t suit our problem well. Instead, we would prefer a method that can detect subgraphs with possible overlaps. Our proposed semi-supervised method overcomes this drawback that many existing graph clustering methods suffered and gives a promising result.

• How to represent protein complexes. Most existing clustering methods assume protein complexes as dense subgraphs, which is not always true for the protein complexes in the PPI networks [19]. In addition, all kinds of topologies present in protein complexes, and tremendous variation of the sizes of protein complexes pose a further problem for identifying the specific topologies. Traditional methods were all non-supervised methods which didn’t fully utilize the properties and features of protein complexes. Here we are trying to use both topological properties and biological properties of protein complexes to represent protein complexes and propose a multi-layer neural network based semi-supervised method to detect the hidden protein complexes.

## Results and discussion

### Data preparation

For our experiments, we built our weighted protein interaction networks from DIP data set [22], which contains 4935 proteins and 14162 interactions. The way to build the weighted network is illustrated in our previous paper [23][24]. In order to evaluate the predicted complexes, the set of real complexes are selected as the benchmarks. This benchmark set is from MIPS [25] and we only select those complexes that contain more than two proteins.

### Validation criterion

In order to study the relative performance of different supervised learning algorithms, we need to define an evaluation criterion that determines if a predicted protein complex matches a complex in benchmark set. In [9], the authors used an overlapping score as the criterion:(1)

where *A* is the predicted complexes, *B* is the true protein complexes, *V_A_* is the set of proteins in the subgraph *A*, and *V_B_* is the set of proteins in the subgraph *B*. In this paper, we use an overlapping threshold of 0*.*20 to determine a match for all experiments. Predicted protein clusters that match one or more true protein complexes with overlapping scores higher than this threshold are identified as “matched clusters,” and the corresponding true complexes are noted as “matched complexes.”

To measure the accuracies of prediction, we calculate prediction, recall and *f*-measure for different algorithms. They are defined as:(2)

where *M_cluster_* is the number of the matched clusters and *P_cluster_* is the number of the predicted clusters, and(3)

where *M_complexes_* is the number of matched complexes and *T_complexes_* is the number of the true complexes. Because smaller size complexes and clusters have high probabilities of occurring by chance and they are not meaningful, here we only consider clusters and complexes whose size is 4 or larger.(4)

where *f*-measure is defined as the harmonic mean of recall and precision. It reflects a combination of precision and recall.

### Comparative evaluation

**Figure 1:**
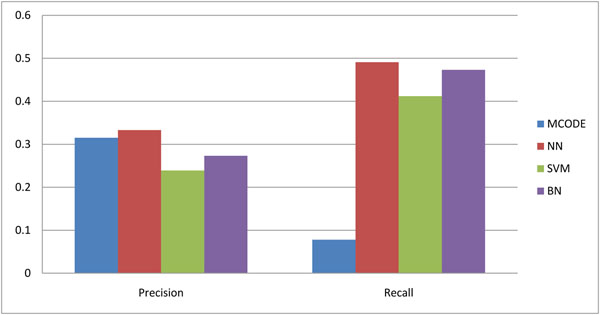
Comparative performance of several approaches in terms of precision and recall for DIP data.

To evaluate the performance of our proposed method, we compared two different supervised learning methods SVM and Bayesian Network (BN) [19] with our method. In addition, we also compared it to a representative non-supervised learning method “MCODE” [9]. “MCODE” is a density based clustering method which is used to find highly interconnected subgraphs in the PPI networks. SVM [26] and Bayesian Network [19] use the same features as our method, showing the effectiveness of the neural network model. The comparison result is presented in Table 1 and in Figure 1. Each method is evaluated by the precision, recall and *f*-measure, separately. As we can see, the proposed method is superior in all measures. The recall rate of our method is around 49%, which is impressive. Our precision is not as high as recall but it is still better than the other existing methods. In addition, our *f*-measure is the highest among all 4 methods. Since many protein complexes are not included in the benchmark set, the predicted protein complexes could be correct protein complexes that are not in the available data. The recall value of MCODE is relatively low compared with other methods. This is because the protein complexes that MCODE found are relatively larger than the clusters found by the other approaches and thus affect the result [19]. In addition, the performances of SVM and Bayesian model are not as good as neural networks; perhaps this is due to the unique way that neural networks train the parameters of the model and the high tolerance to the noisy data.

**Table 1:** Performance comparison of MCODE (Molecular Complex Detection), NN (Neural Network), SVM and BN (Bayesian Network).

Method	Supervised or Not	Precision	Recall	*f*-measure
MCODE	no	0.315	0.078	0.125
NN	yes	0.333	0.491	0.397
SVM	yes	0.239	0.412	0.302
BN	yes	0.273	0.473	0.346

## Conclusions

In this paper, we analyzed and detected protein complexes in protein-protein interaction networks from a different perspective. Instead of using traditional non-supervised algorithms to find dense subgraphs in the PPI networks, we proposed a semi-supervised prediction model with neural network. Unlike previous methods that relied too much on the density of the subgraph, our algorithm utilizes topological and biological features from known protein complexes. With those characterized features, we could represent protein complexes better than the previous methods. Thus a more accurate prediction model can be built upon them. The comparison results show that our algorithm could identify complexes that are missed by other methods. We also have shown that our method achieves better precision and recall rates for the identified protein complexes. In addition, the framework we proposed is easy to be extended in the future. Since obtaining the features of protein complexes and building the prediction model are independent, we could add more representative features of protein complexes in the future work and adopt other similar prediction models that are similar to neural network. In the next step, we hope to find more representative features to formulate protein complexes either from topological manner or biological manner. Also, with more PPI networks of different species becoming available, we could apply the proposed method to the new emerging data sets.

## Methods

While the existing methods identify protein complexes with strong assumptions about their topology (dense subgraph), our proposed method utilizes multiple features that define protein complexes in protein-protein interaction networks. Instead of only assuming the protein complexes as dense subgraphs, we derive several properties from known protein complexes and use these features to search for the new protein complex. Our algorithm first gains the weights for different features from the limited known protein complexes. Then it will assign a score to any subgraph in the graph. With a setup threshold, we could label some of the subgraphs as complexes. With more complexes, we could train the data again and get more suitable weights for the features, thus better prediction model. Recursively, we will find all protein complexes in the PPI network. Compared with the existing method, our proposed model found more accurate protein complexes in the protein-protein interaction network.

### Weighted undirected PPI network

**Figure 2:**
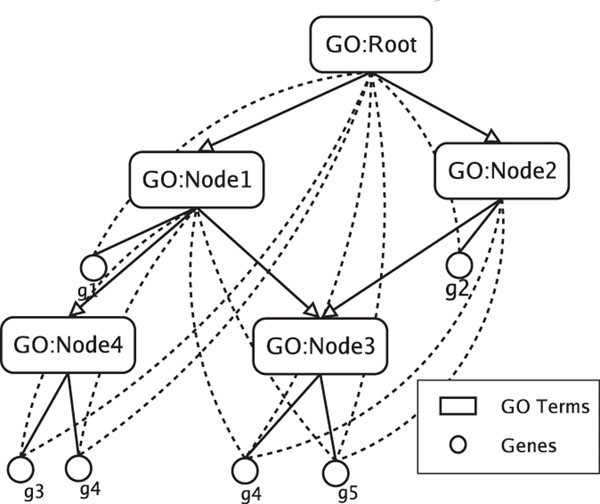
Gene Ontology structure

Many previous works [20] have already pointed out that the PPI networks are very noisy. Table 2 shows the percentage of function-relevant interactions in three proteinprotein interaction data sets, namely, DIP [22], MIPS [25] and BioGrid [27]. An interaction is considered to be function-relevant if the two proteins involved in the interaction have at least one function in common. In this test, we adopt FunCat(version 20070316) [25] in the MIPS database as our annotation categories. From Table 2, we can see that only 30% – 40% observed interactions are relevant in functions. In other words, most of the observed interactions do not share functions. Among those sharing function pairs, some of them share more functions than the others. So there are a lot of false-positive and false-negative interactions in the PPI network and we would better use a weighted graph to represent it rather than the unweighted graph. Throughout the paper, we use a weighted, undirected graph *G* = (*W*, *V*, *E*) to represent the protein interaction network where *V* represents the set of vertices (proteins), *E* represents the set of edges (interactions) and *W* represents the likelihoods for the interactions between vertices. The weights are obtained by using Gene Ontology (GO), which is used for consistent description of genes and gene products. The GO provides a collection of well defined and structured biological terms called GO terms, which is shown in Figure 2. By using GO structure, we could calculate the semantic similarity of different proteins in protein-protein interaction networks, therefore we use them as the weight of the edges. In the previous work [21], we have already successfully obtained a weighted, undirected DIP protein interaction network. Here we use the same network as before.

**Table 2:** The percentage of function-relevant interactions in three protein interaction data sets

Data Set	Total number of interactions	Number of functional-relevant interactions	Percentage
DIP	14162	5216	36.83%
MIPS	13877	4189	30.18%
BioGrids	117675	36446	30.97 %

### Complex features

Choosing the right features to representing the protein complexes is the key issue to our problem. So far, there is a lot of work that has been done in this area. In general, the existing approaches either use properties of nodes and edges or utilize non-trivial substructures. In this paper, we use both topological features of subgraphs and biological features of the proteins in the subgraphs. Most of the features are selected from the prior works on subgraph analysis [19][28][29]. The features that we used are listed in Table 3 and each of the feature types will be briefly discussed in the following.

**Table 3:** The distribution of features.

group ID	group name	number of features
1	Graph density	1
2	Degree statistics	4
3	Edge weight statistics	2
4	Topological change	7
5	Clustering coefficient	3
6	Topological coefficient	3
7	Protein length	2
8	Polarity of amino acids	21

Topological features:

1. Graph density: graph density is defined as , where |*E*| is the number of edges in the graph and |*V*| is the number of vertices in the graph.

2. Degree statistics: these features are calculated from the degree of vertexes in the subgraph. A degree is defined as the number of neighbors of a vertex. Mean degree, variance of degrees, median degree and maximum degree are chosen for degree statistics.

3. Edge weight statistics: we only consider edges with nonzero weights here. Like degree statistics, mean and variance of weights are taken as features.

4. Topological change [28]: This group of features is gained by measuring the topological changes when different cutoffs of the weights are applied to the graph. Topological changes are measures as *T_i_* = (|*E_i_*| – |*E_i_*_+1_|)/|*E_i_*|, where *E_i_* is the number of edges with different cutoffs *i.*

5. Clustering coefficient: the clustering coefficient is a measure of degree to which nodes in a graph tend to cluster together. It is defined as , where |*e*(*i*, *j*)| gives the number of triangles that go through node *v*, whereas *d*(*v*)(*d*(*v*) – 1)*/*2 is the total number of triangles that could pass through node *v.*

6. Topological coefficient [19]: the topological coefficient is a relative measure of the extent to which a protein shares interaction partners with other proteins.

Biological features:

1. Protein length: the number of amino acids in a protein sequence.

2. Polarity of amino acids: the longer and more complementary the binding sites, the majority of which would be polar, of the protein, the stronger the proteins would be bound.

### A two layers feed-forward neural network based model

**Figure 3:**
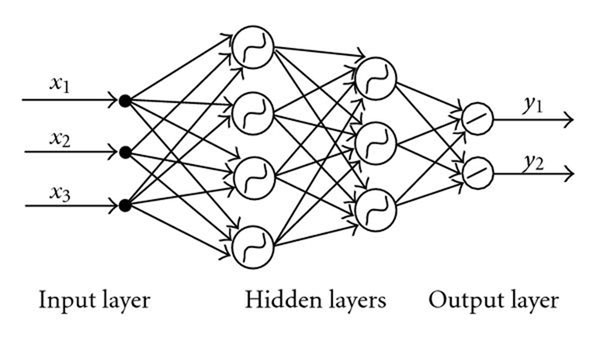
A multiple-layer neural network example

A neural network is a set of connected input/output units in which each connection has a weight associated with it. In the last couple of years, many different kinds of neural networks and corresponding algorithms have been developed. Among the existing algorithms, the **backpropagation** algorithm [30] is the most well known one. The backpropagation algorithm performs learning on a multilayer feed-forward neural network. It iteratively learns a set of weights for prediction of the class label of tuples. Normally a multilayer feed-forward neural network consists of an input layer, multiple hidden layers, and an output layer. To avoid the long learning process, we choose to use the two layers model which contains one input layer, two hidden layers, and one output layer. The PPI network is notorious for its noisy behavior, which contains many false negative connections and false positive connections. Since the neural network is famous for its high tolerance of noisy data, it is an ideal prediction model for our problem [31]. An example of multiple-layer neural network is illustrated in Figure 3. In this example, there are one input layer, two hidden layers and one output layer in the network. **x**(*x*_1_, *x*_2_, *x*_3_) is the input pattern and **y**(*y*_1_, *y*_2_) is the teaching or target vector. During the network training, the signals generated by the output layers are compared with the target vector **y**, and any difference is used in training the weights throughout the network. Some key parameters in the neural network are set as follows. The number of units in the input layer is the number of features that we use (in our case, it is 43). The number of output layers is one in our case, since we only need to know if a subgraph is complex or not, which is a classic bi-class classification. The number of hidden layers is two, and we set 11 units for the first layer and 7 units for the second layer. Those numbers are obtained by experiments starting from a full connected network with a sufficiently large number of nodes to a smaller number of nodes. The activation function for each unit we use is the logistic function. Given the net input *I_j_* to unit *j*, then *O_j_*, the output of unit *j*, is computed as:(5)

Thus, the error of a hidden layer unit *j* is(6)

where *w_jk_* is the weight of the connection from unit *j* to a unit *k* in the next higher layer, and *Err_k_* is the error of *k*. The weights and biases are updated to reflect the propagated errors. Weights are updated by the following equations, where ∆*w_ij_* is the change in weight *w_ij_*:(7)(8)

where *l* is the learning rate. Backpropagation learns using a method of gradient descent to search for a set of weights that fits the training data so as to minimize the mean squared distance between the network’s class prediction and the known target value of the tuples [30]. Biases are updated by the following equations, where ∆*θ_j_* is the change in bias *θ_j_:*(9)(10)

The whole updating process terminates when all ∆*w_ij_* get so small as to be below some specified threshold.

### A semi-supervised learning method for new complexes

Based on the above model, we could use it to evaluate the candidate subgraphs. If the evaluating value exceeds the threshold, the candidate subgraph is predicted to be a complex. So the problem becomes finding subgraphs with high evaluating values in the weighted PPI network. However, as proved in [19], identifying the set of maximally scoring subgraphs in large graph is NP-hard. Thus, heuristic algorithms are needed here. There are several approaches that have already been used to solve this problem, such as hill climbing, simulated annealing, and tabu-search heuristic [32].

**Figure 4:**
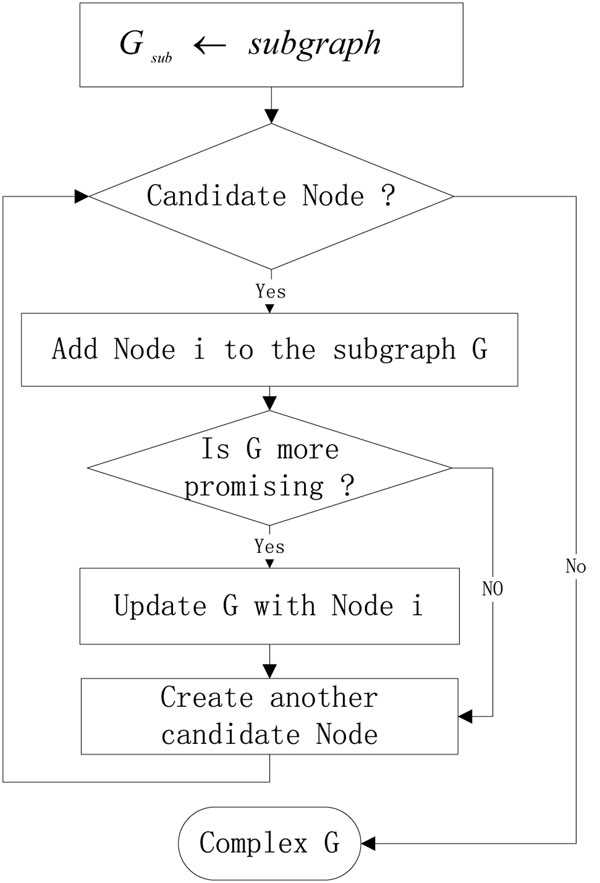
Flow chart of the subgraph generation subroutine

Here we propose a new heuristic method using an evaluation value as the objective function. At the beginning, each cluster starts at a deterministic single node which we call seed node. In our method, we choose the highest weight node as our seed node. The weights of nodes are determined by summing up the weights of the incident edges. Then we add the neighbor nodes of the cluster one by one to the new cluster and the order is based upon their impact on the evaluation score. The adding process stops when there are no more proteins that could be added and our new protein complex is created. The whole subgraph generation routine is illustrated in Figure 4. Also, we keep tracking the overlapping between the existing clusters and the current investing cluster. If the overlapping rate is over a threshold, we will merge those two complexes.

**Figure 5:**
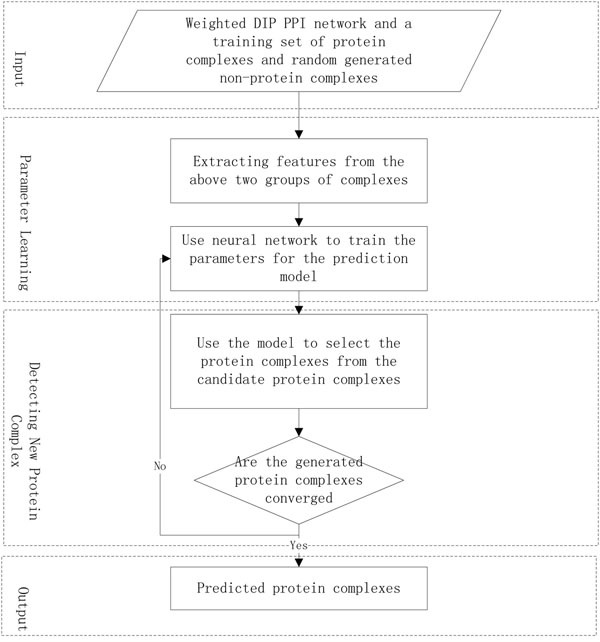
The flow chart of the whole algorithm

The completely proposed algorithm for protein complexes identification is described below and the flow chart of the whole algorithm is shown in Figure 5.

• **Input**

– Weighted DIP PPI network and a training set of protein complexes and randomly generated non-protein complexes.

• **Learning parameters step**

– Extract features from the above two groups of complexes.

– Use neural network to train the parameters for the prediction model.

• **Identifying for complexes**

– Start from the seed nodes, add neighboring proteins of the cluster one by one based on the priority and the impact on the cluster.

– Output the complexes when there is no more proteins to satisfy the criterion given above.

– Use the newly generated complexes to recursively update the parameters of the model in the second step and find the new complex.

• **Output**

– Predict protein complexes.

Our input is the weighted PPI graph and a set of known complexes and non-complexes as training data. The known protein complexes are drawn from MIPS protein complexes and the non-complexes are generated randomly from the DIP protein interaction dataset. First, we use the neural network model to learn model parameters from the training data. Once we get the prediction model, we will start searching for the protein complex. Next, when we have more protein complexes, we recursively train our prediction model and find new protein complexes until there are no more proteins that could be added. The final output complexes are those detected clusters which have a higher evaluation score than the threshold.

## Authors' contributions

LS designed and implemented the algorithm and the framework, analyzed the results and drafted the manuscript. XL participated experiments of the algorithm. AZ coordinated the project and revised the manuscript.

## Competing interests

The authors declare that they have no competing interests.
